# *TLR2* and *TLR4* Polymorphisms Are Not Associated with Dental Caries in Polish Children

**DOI:** 10.3390/ijms25136985

**Published:** 2024-06-26

**Authors:** Marta Milona, Tomasz Olszowski, Izabela Uzar, Krzysztof Safranow, Joanna Janiszewska-Olszowska, Monika Szmidt-Kądys, Hubert Rola, Maciej Sikora, Dariusz Chlubek, Grażyna Adler

**Affiliations:** 1 Department of Hygiene and Epidemiology, Pomeranian Medical University in Szczecin, Powstańców Wlkp. 72, 70-111 Szczecin, Poland; marta.milona@pum.edu.pl (M.M.); tomasz.olszowski@pum.edu.pl (T.O.); 2 Department of General Pharmacology and Pharmacoeconomics, Pomeranian Medical University in Szczecin, Żołnierska 48, 71-210 Szczecin, Poland; izabela.uzar@pum.edu.pl; 3 Department of Biochemistry and Medical Chemistry, Pomeranian Medical University in Szczecin, Powstańców Wlkp. 72, 70-111 Szczecin, Poland; krzysztof.safranow@pum.edu.pl (K.S.); sikora-maciej@wp.pl (M.S.); 4 Department of Interdisciplinary Dentistry, Pomeranian Medical University in Szczecin, Powstańców Wlkp. 72, 70-111 Szczecin, Poland; joanna.janiszewska.olszowska@pum.edu.pl; 5 Department of Conservative Dentistry with Endodontics, Pomeranian Medical University in Szczecin, Powstańców Wlkp. 72, 70-111 Szczecin, Poland; monika.szmidt.kadys@pum.edu.pl; 6 MIL-MED Healthcare Center, Ruta 8, 72-300 Gryfice, Poland; huberter@ymail.com; 7 Department of Maxillofacial Surgery, Hospital of the Ministry of Interior, Wojska Polskiego 51, 25-375 Kielce, Poland; 8 Department of Studies in Anthropogenetics and Biogerontology, Pomeranian Medical University in Szczecin, Żołnierska 48, 71-210 Szczecin, Poland; grazyna.adler@pum.edu.pl

**Keywords:** Toll-like receptors 2 and 4, single nucleotide polymorphisms, dental caries experience, children

## Abstract

The aim of the present study was to analyze the association of the *TLR2* (Toll-like receptor 2 gene) 2258G>A (rs5743708), *TLR4* (Toll-like receptor 4 gene) 896A>G (rs4986790), and *TLR4* 1196C>T (rs4986791) polymorphisms with dental caries in Polish children. The participants, 261 15-year-old children, were divided into two groups: 82 cases (i.e., children with DMFT (Decayed, Missing, and Filled Teeth) index >5, having either moderate or high caries experience, assigned as the “higher” caries experience group) and 179 controls (i.e., children with DMFT ≤ 5, having either low or very low caries experience, assigned as the “lower” caries experience group). Genomic DNA was isolated from buccal swabs, and genotyping was determined by means of real-time PCR (polymerase chain reaction). There were no significant differences in the genotype or allele distributions in all tested SNPs (single nucleotide polymorphisms) between children with “higher” caries experience and those with “lower” caries experience. *TLR4* haplotype frequencies did not differ significantly between cases and controls. In an additional analysis with another case definition applied (subjects with DMFT ≥ 1 were assigned as “cases”, whereas children with DMFT = 0 were assigned as “controls”), no significant differences in the *TLR2* and *TLR4* genotype, allele frequencies, and *TLR4* haplotype frequencies were found between the case and the control groups. The results of the present study broaden our knowledge on the potential genetic factors that might affect caries risk and suggest that *TLR2* rs5743708 and *TLR4* rs4986790 and rs4986791 SNPs are not associated with dental caries susceptibility in Polish children.

## 1. Introduction

Oral diseases (including dental caries) are the most prevalent conditions globally, with untreated dental caries in permanent teeth being the most common health condition according to GBD 2019 study [1]. The WHO estimated the global average prevalence of caries of deciduous teeth as 43% and the global average prevalence of caries of permanent teeth as 29% [2]. Dental caries is a chronic, complex, multifactorial disease that could be classified as a noncommunicable disease (NCD), defined as a biofilm-mediated, sugar-driven condition that leads to the disruption of dental biofilm symbiosis and demineralization of tooth hard tissues [3]. The interaction of many factors contributes to caries development, including environmental factors such as the frequency of toothbrushing, the frequency of sugar-sweetened beverages consumption, breastfeeding, sex, and household income [3,4]. Some studies’ results demonstrate thew significant involvement of genetics in caries susceptibility, with 20–65% of the variation in dental caries experience attributable to genetics [5,6].

In our previous studies, our research group has focused on the association of SNPs (single nucleotide polymorphisms) in selected genes involved in immune response with dental caries (*MBL2*, *MASP2*, *FCN2*, and *FCN1*) [7,8,9]. In the present study, we extend our analysis to other genes involved in immune response, i.e., *TLR2* (Toll-like receptor 2 gene) and *TLR4* (Toll-like receptor 4 gene).

The Toll-like receptor (TLR) family belongs to the so-called pattern-recognition receptors (PRR) and plays important roles in the innate immune response [10,11,12]. There are 11 functionally different TLRs in humans [11]. TLR2 is considered as a key regulator of host immunity, playing a pivotal role in deciding the fate of several microbial and parasitic infections [11]. TLR4 has the unique ability to identify pathogen-associated molecular patterns (PAMPs) from many types of pathogens [11]. TLR2 is involved in Gram-positive bacterial sensing, whereas TLR4 is involved in in Gram-negative bacterial sensing [10,12,13]. TLR2 and TLR4 have been detected in healthy pulp (in the odontoblast cell membrane). TLR2 was shown to be upregulated in odontoblasts beneath caries lesions as compared to odontoblasts beneath healthy dentin, leading to the upregulation of innate immunity effectors (incl. antimicrobial agents) [14]. TLR2 is involved in immune response against cariogenesis [15,16]. TLR4 in human pulp, upon sensing pathogenic bacteria, induces inflammatory cytokine production and activates autophagy to protect the host against bacterial invasion [17].

The association of SNPs in *TLR2* and *TLR4* genes with different infectious diseases (including oral diseases) was examined [11,12,18,19,20,21,22,23,24,25,26,27,28,29]. However, there are very few studies examining the role of the SNPs in these genes in dental caries susceptibility [26,30]. Moreover, the basis for the inclusion of *TLR2* and *TLR4* genes in our genetic association study is the results of Wang and coworkers’ study: both genes are present on the prioritized list of dental caries candidate genes provided by ENDAVOUR, created taking into consideration the magnitude of evidence related to dental caries; *TLR2* gene was ranked 158, whereas *TLR4* gene was ranked 376 out of 960 candidate genes [31].

We hypothesize that genetic variants of *TLR2* and *TLR4* that might potentially influence the TLR2 and TLR4 receptor expression and/or function lead to alterations in cariogenic bacteria recognition, binding, and biofilm formation, thus affecting dental caries susceptibility. The aim of the present study was to analyze the association of the *TLR2* 2258G>A (rs5743708), *TLR4* 896A>G (rs4986790), and *TLR4* 1196C>T (rs4986791) polymorphisms with dental caries in Polish children.

## 2. Results

Our study sample comprised 261 participants (144 males, 117 females). Their median DMFT (Decayed, Missing, and Filled Teeth) index was 4 (range 0–19). The case group (defined as children with DMFT > 5) did not differ significantly from the control group (children with DMFT ≤ 5) by gender (46.3% female in the case group, 44.1% female in the control group, *p* = 0.74). The case group (82 subjects) consisted of 25 children with DMFT = 6, 21 children with DMFT = 7, 19 children with DMFT= 8, 7 individuals with DMFT = 9, 6 participants with DMFT = 10, and 1 participant with DMFT = 11, DMFT = 12, DMFT = 13, and DMFT = 19, respectively. The control group (179 subjects) consisted of 32 participants with DMFT = 0, 27 participants with DMFT = 1, 34 children with DMFT = 2, 25 children with DMFT = 4, and 27 with DMFT = 5.

The distributions of all of the tested SNPs, i.e., *TLR2* 2258G>A (rs5743708), *TLR4* 896A>G (rs4986790), and *TLR4* 1196C>T (rs4986791), showed no variant homozygotes, and they did not deviate significantly (*p* > 0.5) from the Hardy–Weinberg equilibrium (HWE).

**Figure 1:**
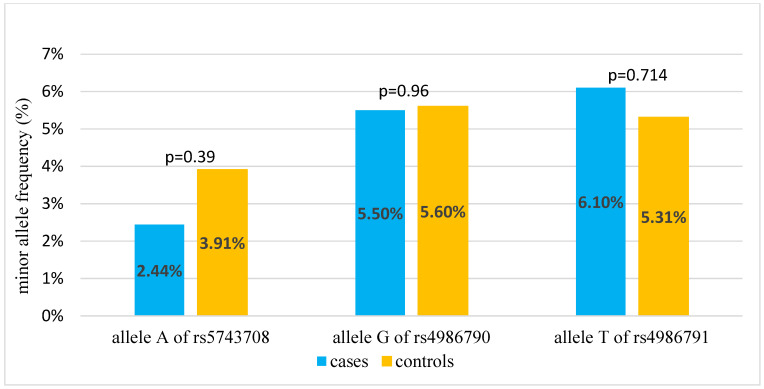
Minor allele frequencies of analyzed SNPs in the case and control group.

The genotype and allele frequencies of the analyzed SNPs of *TLR2* and *TLR4* genes in Polish children are presented in Table 1 and Table 2 and Figure 1.

There were no significant differences in the genotype or allele distributions in all tested SNPs between children with “higher” caries experience (having DMFT index > 5) and those with “lower” caries experience (DMFT ≤ 5).

The analysis of the association between the *TLR2* and *TLR4* SNPs and caries susceptibility, assessed using dominant and recessive genetic models, revealed no statistically significant association between *TLR2* (rs5743708) and *TLR4* (rs4986790 and rs4986791) gene polymorphisms and susceptibility to dental caries (Table 1 and Table 2).

A strong linkage disequilibrium between *TLR4* rs4986790 and rs4986791 SNPs was found (Lewontin’s D’ = 0.963, r^2^ = 0.928).

Four *TLR4* haplotypes (SNPs 896A>G,1196C>T) were identified in the examined subjects, AC, GT, GC, AT, with frequencies of 94.2%, 5.4%, 0.2% and 0.2%, respectively.

*TLR4* haplotype frequencies did not differ significantly between the cases (children with DMFT > 5) and the controls (children with DMFT ≤ 5) (Table 3).

In an additional analysis with another case definition applied (subjects with DMFT ≥ 1 assigned as cases compared to children with DMFT = 0 assigned as controls), no significant differences in the *TLR2* and *TLR4* genotype, allele frequencies, or *TLR4* haplotype frequencies were found between the cases and the controls (*p* > 0.1).

## 3. Discussion

Polymorphisms in *TLR2* and/or *TLR4* genes and protein concentrations of TLR2 and TLR4 have been analyzed in several infectious and/or inflammatory diseases, such as tuberculosis [19], severe invasive infections by *Streptococcus pneumoniae* and *Neisseria menigitidis* [18,21], sepsis [20], infection by *Legionella pneumophila* [11], leprosy [11], infective endocarditis [32], Lyme disease [33], urinary tract infections [34], cytomegalovirus infection [23], respiratory syncytial virus infection [11], HIV infection [22], and malaria [11].

Some studies have demonstrated the involvement of TLR2 and TLR4 in the pathogenesis of oral diseases, such as chronic periodontitis and dental caries [11,15,16,24,25,26,35,36,37,38].

TLR2 and TLR4 are involved in the immune response against bacterial pathogens (both Gram-positive and Gram-negative bacteria) in cariogenesis and in periodontitis [10,16,25,36,39,40,41]. In a study by Malekafzali et al., the elevated salivary concentration of TLR2 in a early childhood caries (ECC) group compared to a caries-free group was demonstrated, suggesting the involvement of TLR2 in the pathogenesis of early childhood caries [15]. Zhao et al. found a significantly increased concentration of soluble TLR2 in the saliva of children with active caries compared to the saliva of children free from caries [16]. Liu et al. demonstrated significantly higher expression of TLR4 in dental pulp tissue affected by caries as compared to dental pulp tissue unaffected by caries [38].

To our knowledge, this is the third report in which the role of *TLR* gene polymorphisms on dental caries susceptibility was addressed. However, the authors of the first study [26] examined a different ethnic group (i.e., subjects from Turkey), a different age group (25–44 years), different *TLR2* SNPs, only one SNP in the *TLR4* gene, and a different caries phenotype as compared to our study. The authors of the second report analyzed different SNPs of *TLR4* (as compared to our study) and no SNPs in *TLR2* [30].

We found a lack of association of alleles, genotypes of *TLR2* and *TLR4* SNPs, and *TLR4* haplotypes with dental caries in Polish children, suggesting that these gene variants do not affect the caries risk (susceptibility).

The results of our study are consistent with the results of the study of Yildiz Telatar et al., who demonstrated a lack of significant differences in *TLR2* (rs121917864) and *TLR4* (rs4986790) gene polymorphisms between the low and high caries risk groups in individuals aged 25–44 years from Turkey [26]. Raivisto et al. found no statistical association between another SNP in the *TLR4* gene, i.e., rs11536889, and initial caries lesions in Finnish adolescents aged 15–17 years old [30].

It is also worth mentioning the results of the study by Alotaibi and coworkers, who conducted a genome-wide association study (GWAS) of dental caries in a large multiethnic population; the authors found that in the permanent dentition, there were no SNPs reaching a genome-wide significance level [42]. Shungin and coworkers in their genome-wide association study and meta-analysis identified 47 novel, conditionally independent genetic risk loci for dental caries with at least one variant meeting the threshold for genome-wide significance (*p* < 5 × 10^−8^) in unconditional results and following a stepwise selection procedure. The authors integrated GWAS results with external sources of expression data, creating a prioritized list of 221 genes that may be involved in dental caries pathogenesis. However, *TLR2* and *TLR4* genes were not present on that list [43].

Our study has some limitations. These include the relatively small sample size and the lack of measurements of TLR2 and TLR4 protein concentrations. Another limitation of this study is that the analysis was restricted to polymorphisms of two genes. In future gene association studies of dental caries, other genes involved in Toll-like receptor-mediated pathogen recognition and/or regulation, such as *TLR1* (Toll-like receptor 1 gene), *TLR6* (Toll-like receptor 6 gene), and *MD-2* (myeloid differentiation-2 gene), should also be considered [44].

## 4. Material and Methods

This study constitutes the continuation of the research project entitled “Polymorphisms of Selected Genes Involved in Immune Response Mechanisms and Dental Caries in Children” (Department of Hygiene and Epidemiology, Pomeranian Medical University in Szczecin) [7,9]. The case–control study design was applied in our study. A total of 261 15-year-old children from Szczecin city were enrolled in our study, as described previously [9]. The study protocol was approved by the Bioethical Committee of Pomeranian Medical University. The study was conducted in accordance with the principles of the Declaration of Helsinki, and both participants and their caregivers gave written informed consent [9].

The mean fluoride concentration in tap water in Szczecin ranges from 0.11 to 0.25 mg/L (data received from the Municipal Sanitary Epidemiological Inspectorate). The vast majority of study participants declared that they brushed their teeth at least twice daily. More advanced tests of the oral material were not performed due to the difficulty in obtaining consent for invasive procedures in children.

Dental caries diagnosis was established based on the presence of cavitation according to recommendations of the WHO [45]. For the purpose of the inspection of dental surfaces for open cavities, a dental lamp and a mirror were used. Clinical visual–tactile examinations were performed with the use of a standard blunt dental probe and a mirror. Dental caries was recorded when a cavity could be diagnosed e.g., enamel breakdown could be observed following the removal of the eventual plaque covering the lesion [46]. The DMFT index (the sum of decayed, missing due to caries and filled teeth) was provided for every participant. Dental caries experience level was evaluated according to Vieira and coworkers [47], taking into consideration the subjects age and DMFT scores. The participants were divided into two groups: 82 cases (i.e., children with DMFT > 5, having either moderate or high caries experience, assigned as the “higher” caries experience group) and 179 controls (i.e., children with DMFT ≤ 5, having either low or very low caries experience, assigned as the “lower” caries experience group). In addition, the analyzed SNP distribution was also compared between the 229 children with caries experience (with DMFT > 0) and 32 children with no caries experience (having DMFT = 0).

Genomic DNA was isolated from buccal swabs by means of standard procedures using the QIAamp DNA Mini Kit (Qiagen, Hilden, Germany). Two *TLR4* SNPs, rs4986790 and rs4986791, and one *TLR2* SNP, rs5743708, were selected as per the NCBI SNP database (https://www.ncbi.nlm.nih.gov/snp/, accessed on 23 June 2024). Genotypes were determined by means of real-time PCR using the StepOneTM Real-Time PCR System (Roche Diagnostics, Warsaw, Poland), the TaqPath ProAmp Master Mix, and commercial pre-designed TaqMan SNP Genotyping Assays (Assay ID: C_11722238_20, C_11722237_20 and C_27860663, respectively) (Thermo Fisher Scientific, Waltham, MA, USA). Samples were first heated at 95 °C for 10 min before amplification as follows: 40 cycles of two-step PCR at 95 °C for 15 s and 60 °C for 1 min. The data were analyzed with Taq Man Genotyper Software v. 1.0.1. (Thermo Fisher Scientific, Waltham, MA, USA). For quality control purposes, approximately 10% of the samples were re-genotyped in a blinded fashion and the same results were obtained.

The results of this study were analyzed statistically with the use of the Statistica 12 software (StatSoft, Kraków, Poland). Genotype and allele frequencies were determined by means of the direct counting method. The chi-square test was used to compare the frequencies of *TLR2* and *TLR4* genotypes, alleles, and *TLR4* haplotypes between groups. The statistical analysis adopted a significance level (α) of 0.05 for the test. The Haploview 4.2 software (Broad Institute, Cambridge, MA, USA) was used for the linkage disequilibrium analysis. Our study with 82 cases and 179 controls had sufficient statistical power to detect with 80% probability true differences in allele frequencies between the groups corresponding to the odds ratio (OR) for the association of moderate/high caries experience with variant alleles of each SNP equal to 2.88 (positive association) or 0.01 (negative association) for rs5743708, 2.53 or 0.13 for rs4986790, and 2.57 or 0.11 for rs4986791.

## 5. Conclusions

The results of the present study broaden our knowledge on the potential genetic factors that might affect caries risk and suggest that *TLR2* rs5743708 and *TLR4* rs4986790 and rs4986791 SNPs are not associated with dental caries susceptibility. However, further studies conducted in other populations/ethnic groups examining other SNPs are needed to test the consistency of our findings in order to draw definitive, final conclusions regarding the association between *TLR2* and *TLR4* SNPs and dental caries. It is also recommended to conduct studies analyzing the role of *TLR2* and *TLR4* gene–environment interactions in dental caries susceptibility. Furthermore, detailed investigations of functional polymorphisms in dental caries candidate genes will allow researchers to construct caries prediction models enabling us to identify high-caries-risk groups among children in order to apply preventive or therapeutic interventions. The knowledge of genetic factors contributing to caries risk/resistance contributes to the development of “precision dentistry”.

## Figures and Tables

**Table 1:** Genotype and allele frequencies of analyzed SNP in the *TLR2* gene in Polish children (DMFT = 5 as a cut-off value).

SNP (Reference Sequence), Genotype/Allele	Children with Moderate/High Caries Experience (DMFT > 5), N = 82 n (%)	Children with Low/Very Low Caries Experience (DMFT ≤ 5), N = 179 n (%)	*p* Value ^a^	OR (95%CI) ^b^	Chi^2^
*TLR2* 2258G>A (rs5743708)					
GG	78 (95.12)	165 (92.18)	0.38	–	0.76
GA	4 (4.88)	14 (7.82)			
AA	0 (0)	0 (0)			
GG + GA	82 (100)	179 (100)	–	–	
AA	0	0			
GG	78 (95.12)	165 (92.18)	0.38	0.60 (0.19–1.89)	0.76
AA + GA	4 (4.88)	14 (7.82)			
G	160 (97.56)	344 (96.09)	0.39	0.61 (0.199–1.895)	0.73
A	4 (2.44)	14 (3.91)			

^a^ Chi-square test; ^b^ odds ratio (OR) and 95% confidence interval (95%CI) for “case” vs. “control” children and “lower” vs. “upper” genotype or allele.

**Table 2:** Genotype and allele frequencies of analyzed SNPs in the *TLR4* gene in Polish children (DMFT = 5 as a cut-off value).

SNP (Reference Sequence), Genotype/Allele	Children with Moderate/High Caries Experience (DMFT > 5), N = 82 n (%)	Children with Low/Very Low Caries Experience (DMFT ≤ 5), N = 179 n (%)	*p* Value ^a^	OR (95%CI) ^b^	Chi^2^
*TLR4* 896A>G (rs4986790)					
AA	73 (89.02)	159 (88.83)	0.96	–	0.002
AG	9 (10.98)	20 (11.17)			
GG	0	0			
AA + AG	82 (100)	179 (100)	–	–	
GG	0	0			
AA	73 (89.02)	159 (88.83)	0.96	0.98 (0.43–2.26)	0.002
GG + AG	9 (10.98)	20 (11.17)			
A	155 (94.5)	338 (94.4)	0.96	0.98 (0.44–2.20)	0.002
G	9 (5.5)	20 (5.6)			
*TLR4* 1196C>T (rs4986791)					
CC	72 (87.8)	160 (89.39)	0.706	–	0.14
CT	10 (12.2)	19 (10.61)			
TT	0	0			
CC + CT	82 (100)	179 (100)	–	–	
TT	0	0			
CC	72 (87.8)	160 (89.39)	0.706	1.169 (0.5179–2.6414)	0.14
TT + CT	10 (12.2)	19 (10.61)			
C	154 (93.9)	339 (94.69)	0.714	1.159 (0.526–2.55)	0.13
T	10 (6.10)	19 (5.31)			

^a^ Chi-square test; ^b^ odds ratio (OR) and 95% confidence interval (95%CI) for “case” vs. “control” children and “lower” vs. “upper” genotype or allele.

**Table 3:** *TLR4* haplotype frequencies in Polish children with moderate/high caries experience versus those with low/very low caries experience.

*TLR4* Haplotypes +896A>G/+1196 C>T	Haplotype frequency (%)		*p* Value ^a^	Chi^2^
	Children with Moderate/High Caries Experience (DMFT > 5) N = 82	Children with Low/Very Low Caries Experience (DMFT ≤ 5) N = 179		
AC	93.9%	94.4%	0.82	0.054
GT	5.5%	5.3%	0.93	0.007
GC	0%	0.3%	0.50	0.458
AT	0.6%	0%	0.14	2.183

^a^ Chi-square test.

## Data Availability

The data that support the findings of this study are available from the corresponding author upon reasonable request.
